# It's time to take the psychology of biological time into account: speed of driving affects a trip's subjective duration

**DOI:** 10.3389/fpsyg.2014.01028

**Published:** 2014-09-16

**Authors:** Hedderik van Rijn

**Affiliations:** Experimental Psychology, University of GroningenGroningen, Netherlands

**Keywords:** interval timing, real-world tasks, perceived speed, driving, time perception, temporal cognition, time dilation, subjective time

The last decades have seen a surge in research into interval timing (for recent reviews, see Merchant et al., 2013; Wittmann, 2013; Allman et al., 2014; van Rijn et al., 2014), with some work focussing on the more abstract mechanisms underlying interval timing (e.g., Taatgen et al., 2007) or the role of cognitive faculties such as memory and decision processes on interval timing tasks (e.g., Taatgen and van Rijn, 2011; Shi et al., 2013), but a large proportion of the work focuses the neural substrates of human (e.g., Kononowicz and van Rijn, 2011; Wiener et al., 2012; Kononowicz and Van Rijn, 2014) and animal (e.g., Díaz-Mataix et al., 2013; Bartolo et al., 2014; Cheng et al., 2014) timing processes. Based on this work, we are getting closer to unraveling the biological mechanisms underlying interval timing.

Interestingly, although an accurate sense of the passing of time at short timeframes (e.g., less than a couple of seconds) is an important building block in many cognitive tasks, many papers—including some of our own—still use rather hackneyed examples such as the timing involved in deciding whether to brake when a traffic light turn yellow to stress the importance of interval timing in every days tasks. Although timing is obviously involved in such real world tasks, the complexities of these tasks are far removed from the simple paradigms using in interval timing studies. For one, in many real-world timing tasks, the temporal stimulus has a direct relevance for the person doing the timing, whereas in most laboratory experiments the participant is asked to time an external stimulus—a distinction which can be compared to a first vs. a third-person perspective on time. These discrepancies make it difficult to generalize from the highly specific experiments in the lab to the psychology of timing as observed in the real world (see also Matthews and Meck, 2014), with a possible exception for studies on target interception (for a review, see Merchant and Georgopoulos, 2006). Although literature does list a number of papers in which interval timing aspects are being studied in the real world (e.g., Ten Bosch et al., 2005; Miller and Fu, 2007), those studies are often not focused on the mechanisms underlying timing, or require large databases with naturalistic data. To ensure that interval timing does not follow the path of some other fields of science—where no one apart from the researchers active in that field remember why a particular phenomenon was interesting enough to study—we should study interval timing not just in artificial tasks that are specifically created to test a particular phenomenon, but also in tasks that have a clear analog to complex, real-life interval timing tasks.

To demonstrate the viability of this approach, below I will discuss a simple experiment based on a prototypical interval-timing paradigm (e.g., Kononowicz and Van Rijn, 2014) set in the context of the evaluation of the speed of a car from a first-person perspective (e.g., as driver or co-driver).

Theories of human time perception typically assume a clock that provides temporal information to decision processes (van Rijn et al., 2011). Although the exact formulation of this clock is still subject of discussion (see for a review, van Rijn et al., 2014), most theories assume that the information emitted by this clock is relatively stable over time. However, both endogenous (e.g., neurochemical fluctuations, Coull et al., 2011) and exogenous manipulations (e.g., contextual changes, van Rijn and Taatgen, 2008; Lui et al., 2011; or manipulations of expectancy, e.g., Tse et al., 2004; see Grondin, 2001, for an extensive review) affect interval timing. For example, if the display duration of a moving stimulus has to be estimated, a positive correlation is found between speed and the estimated display duration (e.g., Brown, 1995; Kline and Reed, 2012, see also Roelofs and Zeeman, 1951–1952), if a stimulus is perceived to move toward an observer it is perceived as having a longer duration than when the same stimulus is presented as a static image or is perceived to be moving away (e.g., van Wassenhove et al., 2008; New and Scholl, 2009; Wittmann et al., 2010), or if the environment in which a temporal stimulus is presented moves faster, the duration of the temporal stimulus is overestimated (e.g., Mate et al., 2009) compared to static or slower moving environments. The interpretation of types of studies into the subjective dilation of time can be roughly summarized as faster movement, or movement toward rather than moving away from the observer, yielding a faster ticking internal clock, resulting in the subjective duration of the stimulus lengthening. These effects are most likely driven by early visual processes that detect the (number of) changes in a display (Droit-Volet and Wearden, 2002), possibly as early as the primary visual cortex (Kanai et al., 2006). Typically, these types of studies ask participants to estimate the duration of stimuli that move on the screen, demonstrating that the subjective perception of time can be affected by perceiving third-person movement. Here we address the question whether simulated first-person perspective movement also affects the estimation of time. If these effects generalize to first-person perspective, this might have direct consequences for naturalistic, real-life settings such as the subjective evaluation of driving speed and speed limits. That is, faster speeds result in faster movement of the scenery, which might result in a lengthening of subjective time. To offset these effects, drivers might be tempted to drive faster, giving rise to more speed violations and a potential positive correlation between absolute speed limit and dissatisfaction with these limits—demonstrating that modulations of interval timing processes can have significant real world implications.

The study was designed to test whether time dilation effects due to moving stimuli generalized to first-person perspective, and what the consequences are on the perceived duration of (short) drives. Using the driving simulator Distract-R (http://cog.cs.drexel.edu/distract-r/; see Salvucci, 2009), we recorded a video clip of several minutes of a car driving at 100 km/h, see Panel A of Figure 1. Participants were acquainted with the standard duration of 2.5 s by presenting them five unique 2.5 s segments of the video. After this presentation, participants were trained on reproducing this interval during 30 reproduction trials. Hereto unique segments of the video where started, and participants were asked to indicate when the just perceived duration had passed by pressing a key, after which feedback (see Kononowicz and van Rijn, 2011, for details) was provided. The experimental phase consisted of 200 temporal generalization trials, in which participants were presented video segments of either 2.3, 2.4, 2.6, or 2.7 s and which they had to categorize as either shorter or longer than the learned duration. Critically, the video segments where either taken from the recorded video, or from video that was slowed down to represent 50 or 75 km/h, or that was sped up to 125 or 150 km/h. Video segments were randomly assigned to speed conditions. Figure 1B shows the average proportions of long responses (38 individuals participated, 3 removed for not following instructions, all participants associated with the University of Groningen, ethical approval #12242-NE, Ethical Committee Psychology) separately for each simulated car-speed condition (solid lines). The five lines indicate that participants are sensitive to the duration manipulation, with shorter durations less often categorized as long than longer durations. Moreover, the curves are vertically ordered in line with the depicted speed, indicating that video clips depicting faster speeds were more likely to be categorized as long. A binomial linear mixed effect model (glmer from the lme4 package 1.1–6 in R 3.0.2 using logit as link-function and estimating fixed-effects parameters and random effects in a linear predictor using maximum likelihood), with centered fixed effects for speed (i.e., expressing speed as −50, −25, 25, and 50) and for duration (−0.2, −0.1, 0.1, 0.2) and a random intercept, and separate random slopes for speed and duration per participants, confirmed the effects of duration and speed (β_speed_ = 0.0187, *SE* = 0.0025, *z* = 7.573 and β_duration_ = 3.5432, *SE* = 0.4569; *z* = 7.754 respectively, *p*s < 0.0001), the interaction between duration and speed was not significant (β_speed × duration_ = 0.0064, *SE* = 0.0052, *z* = 1.251, *p* = 0.21), nor was the intercept of the model (β_intercept = −0.0756_, *SE* = 0.0779, *z* = −0.971, *p* = 0.33). Indeed, model comparisons indicate that the addition of the interaction was not warranted [χ^2^_(1)_ = 1.55, *p* = 0.2129].

**Figure 1:**
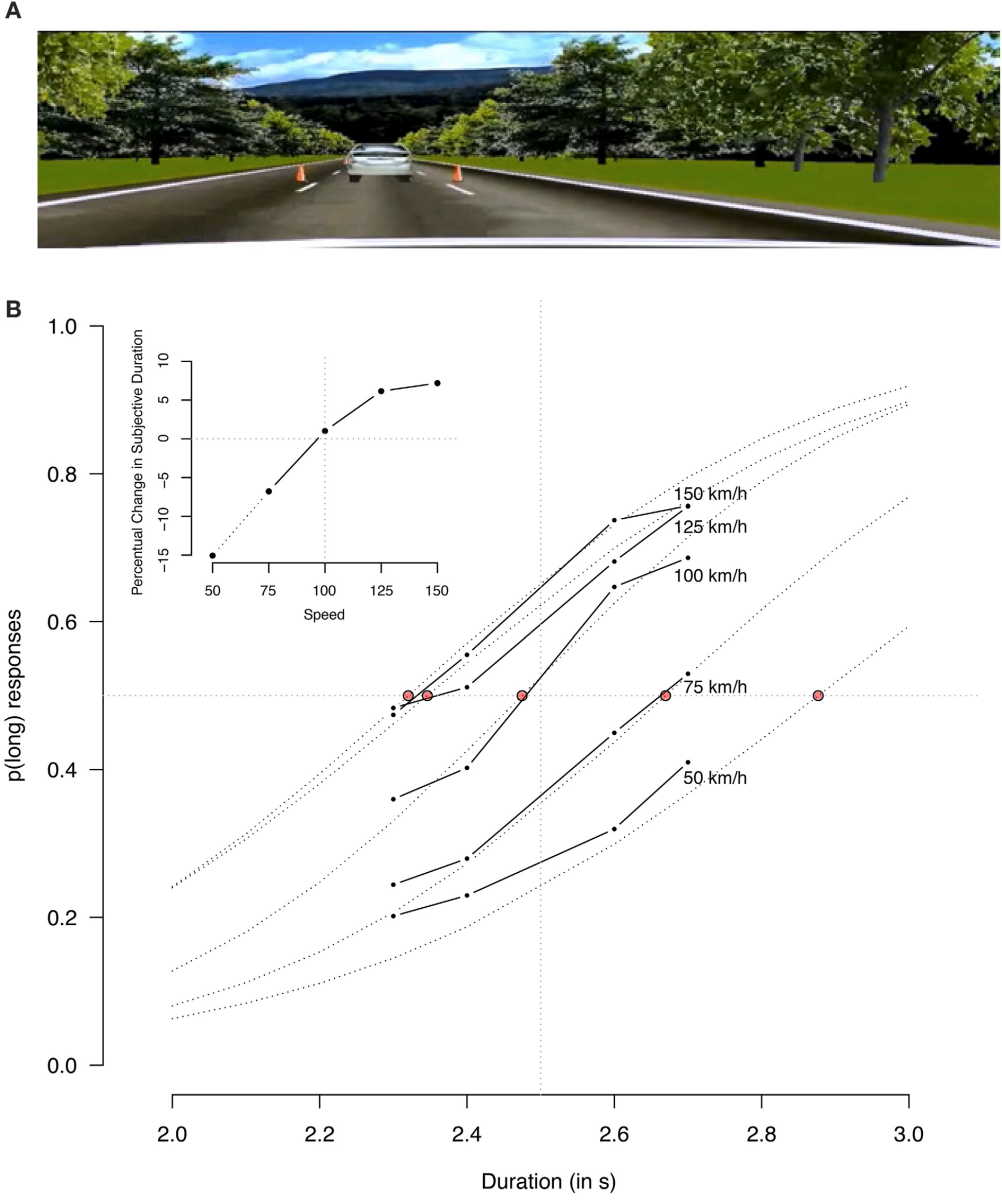
**(A)** Depicts a still from the video of which the participants had to estimate whether the duration was shorter or longer than the standard. **(B)** Presents the proportion of long categorizations for the five speed conditions and the four experimental durations. The inset depicts the relative dilation for the five speed conditions, derived from the red dots representing the point of subjective equality.

This initial model assumes that there is a linear effect of speed. Because Figure 1 suggests a nonlinear mapping of speed on probability long responses, we also conducted an analysis with speed as a factorial variable (reference: 100 km/h). Although this increases the complexity of the model, the fit is sufficiently improved to select this more complex model [χ^2^_(21)_ = 50.684, *p* = 0.0003]. As before, the inclusion of the interaction is not warranted [χ^2^_(4)_ = 3.3126, *p* = 0.5069]. The estimated effect size of the intercept does not deviate from zero (β = 0.10391, *SE* = 0.09179, *z* = 1.132, *p* = 0.258), indicating that no change in speed does not significantly affect the subjective perception of time. The estimates for the other four speed conditions are in the expected direction (β_−50_ = −1.28323, *SE* = 0.20088; β_−25_ = −0.72023, *SE* = 0.11840; β_25_ = 0.41170, *SE* = 0.09786; β_50_ = 0.53864, *SE* = 0.11022; |z| > 4.2, *p* < 0.0001), and the estimated duration effect is similar to the previous model (β = 3.56750, *SE* = 0.46144, *z* = 7.731, *p* < 0.0001). The estimated psychometric curves resulting from this last model are plotted in Figure 1B as dotted lines. Note that these lines are extended beyond the direct measured data based on the estimates of the binomial linear mixed effect model.

The circles drawn around the intersections between the fitted speed-specific psychometric functions and the p(long) = 0.5 line represent the point of subjective equality (PSE), that is, at what point in the duration of the movie clip (would have) felt as long as the standard duration of 2.5 s. Note that as the 50% decrease condition resulted in few long categorizations, the estimated PSE is extrapolated from the estimated psychometric function. On the basis of these data, the inset depicts the dilation as a function the speed of the video. At 100 km/h, participants' estimations are quite accurate, with a dilation of only 1.0%. For the 50% increase in speed condition, the subjective lengthening relative to the baseline condition of 100 km/h is 7.2%, and for the 25% increase in speed 6.2%. For the 25% decrease in speed, the subjective shortening is 6.8%, and for the 50% decrease in speed, the extrapolated estimate is a subjective shortening of 15%. Over the four conditions, the average effect of a 10% increase or decrease of speed on time is a dilation or contraction of 2.4%, indicating that about one fourth of the objective advantage of increased speed limits is canceled out by subjective lengthening of time. These results demonstrate that the effect of the perception of movement on interval timing extends to movement in first-person perspective, and can also be observed in naturalistic, yet well controlled conditions.

These results are in line with the third-person perspective studies on the effect of movement on time. That is, the faster the speed of the car in the video, the more context changes were perceived in the video, aligning nicely with the hypothesis that the number of visual changes drives temporal dilation effects. Although tested in a different context, this explanation find corroborative support in a study (Antonson et al., 2009) on the effect of landscapes on preferred speed in car simulators, with landscapes richer in details (i.e., forests compared to open spaces) associated with slower preferred speeds. Although this effect is typically explained by other, higher-level factors, our study suggests that the rich detail landscapes might cause internal time to run faster due to the higher number of changes, causing participants to drive slower to keep their subjective speed at comfortable levels. A similar finding that links the subjective perception of speed with interval timing is reported in Rudin-Brown (2004) who has shown that eye height of a driver affects preferred speed, with drivers seated higher preferring faster speeds caused by the subjectively slower movement of the outside world. As suggested by a reviewer, an elegant test that could provide further links between laboratory tasks and task-settings with higher external validity is to compare conditions with meaningful semantic visual context versus phase-scrambled movies, which would make the current experiment better comparable to laboratory studies in which semantically irrelevant movement is provided. Comparing these results will allow us to assess directly whether the first-person perspective in a meaningful context affects subjective interval timing.

To summarize, this study shows that the temporal dilation effects observed in lab-studies on interval timing, including but not limited to the phenomena discussed earlier (Eagleman, 2008), have real world consequences: if a driver is used to driving at 100 km/h, and is suddenly allowed to drive 130 km/h, the dilation of time will result in an internal experience of approximately 123 km/h. Compensating for this subjective discrepancy will cause speeding, whereas adherence to the speed limit will cause the driver to perceive a discrepancy with the enforced limits and his or her internal evaluation of speed. Moreover, this study also shows that generalizing findings from the lab is possible, and that appealing examples can be found that demonstrate the relevance for interval timing in real-world settings.

## Conflict of interest statement

The author declares that the research was conducted in the absence of any commercial or financial relationships that could be construed as a potential conflict of interest.

## References

[B1] Allman M. J. Teki S. Griffiths T. D. Meck W. H. (2014). Properties of the internal clock: first- and second-order principles of subjective time. Annu. Rev. Psychol. 65, 743–771 10.1146/annurev-psych-010213-115117 24050187

[B2] Antonson H. Maringrdh S. Wiklund M. Blomqvist G. (2009). Effect of surrounding landscape on driving behaviour: a driving simulator study. J. Environ. Psychol. 29, 493–502 10.1016/j.jenvp.2009.03.005

[B3] Bartolo R. Prado L. Merchant H. (2014). Information processing in the primate basal ganglia during sensory-guided and internally driven rhythmic tapping. J. Neurosci. 34, 3910–3923 10.1523/JNEUROSCI.2679-13.2014 24623769 PMC6705277

[B4] Bosch L. T. Oostdijk N. Boves L. (2005). On temporal aspects of turn taking in conversational dialogues. Speech Commun. 47, 80–86 10.1016/j.specom.2005.05.009

[B5] Brown S. W. (1995). Time, change, and motion: the effects of stimulus movement on temporal perception. Percept. Psychophys. 57, 105–116 10.3758/BF03211853 7885802

[B6] Cheng R. K. Jesuthasan S. J. Penney T. B. (2014). Zebrafish forebrain and temporal conditioning. Philos. Trans. R. Soc. Lond. B Biol. Sci. 369, 1471–2970 10.1098/rstb.2012.0462 24446496 PMC3895987

[B7] Coull J. T. Cheng R.-K. Meck W. H. (2011). Neuroanatomical and neurochemical substrates of timing. Neuropsychopharmacology 36, 3–25 10.1038/npp.2010.113 20668434 PMC3055517

[B8] Díaz-Mataix L. Ruiz Martinez R. C. Schafe G. E. LeDoux J. E. Doyère V. (2013). Detection of a temporal error triggers reconsolidation of amygdala-dependent memories. Curr. Biol. 23, 467–472 10.1016/j.cub.2013.01.053 23453952 PMC3606686

[B9] Droit-Volet S. Wearden J. (2002). Speeding up an internal clock in children? Effects of visual flicker on subjective duration. Q. J. Exp. Psychol. B 55, 193–211 10.1080/02724990143000252 12188524

[B9a] Eagleman D. M. (2008). Human time perception and its illusions. Curr. Opin Neurobiol. 18, 131–136 10.1016/j.conb.2008.06.002 18639634 PMC2866156

[B10] Grondin S. (2001). From physical time to the first and second moments of psychological time. Psychol. Bull. 127, 22–44 10.1037/0033-2909.127.1.22 11271754

[B11] Kanai R. Paffen C. L. E. Hogendoorn H. Verstraten F. A. J. (2006). Time dilation in dynamic visual display. J. Vis. 6:8 10.1167/6.12.8 17209745

[B12] Kline S. R. Reed C. L. (2012). Contextual influences of dimension, speed, and direction of motion on subjective time perception. Atten. Percept. Psychophys. 75, 161–167 10.3758/s13414-012-0370-4 22976846

[B13] Kononowicz T. W. van Rijn H. (2011). Slow potentials in time estimation: the role of temporal accumulation and habituation. Front. Integr. Neurosci. 5:48 10.3389/fnint.2011.00048 21949505 PMC3171873

[B14] Kononowicz T. W. Van Rijn H. (2014). Decoupling interval timing and climbing neural activity: a dissociation between CNV and N1P2 amplitudes. J. Neurosci. 34, 2931–2939 10.1523/JNEUROSCI.2523-13.2014 24553934 PMC6608524

[B15] Lui M. A. Penney T. B. Schirmer A. (2011). Emotion effects on timing: attention versus pacemaker accounts. PLoS ONE 6:e21829 10.1371/journal.pone.0021829 21799749 PMC3140483

[B16] Mate J. Pires A. C. Campoy G. Estaun S. (2009). Estimating the duration of visual stimuli in motion environments. Psicológica 30, 2878–2300

[B17] Matthews W. J. Meck W. H. (2014). Temporal *Perception*: *Taking* the *Good With* the *Bad*. Wiley Interdisciplinary Reviews: Cognitive Science. 10.1002/wcs.1298 25210578 PMC4142010

[B18] Merchant H. Georgopoulos A. P. (2006). Neurophysiology of perceptual and motor aspects of interception. J. Neurophysiol. 95, 1–13 10.1152/jn.00422.2005 16339504

[B19] Merchant H. Harrington D. L. Meck W. H. (2013). Neural basis of the perception and estimation of time. Annu. Rev. Neurosci. 36, 313–336 10.1146/annurev-neuro-062012-170349 23725000

[B20] Miller S. M. Fu W.-T. (2007). The role of temporal sequence learning in guiding visual attention allocation, in Presented at the Proceedings of the Human Factors and Ergonomics Society 51th Annual Meeting (Baltimore, MD: SAGE Publications), 1368–1372

[B21] New J. J. Scholl B. J. (2009). Subjective time dilation: spatially local, object-based, or a global visual experience? J. Vis. 9, 4.1–4.11 10.1167/9.2.4 19271914

[B22] Roelofs C. O. Zeeman W. P. C. (1951–1952). Influence of different sequences of optical stimuli on the estimation of duration of a given interval of time. Acta Psychol. (Amst). 8, 89–128 10.1016/0001-6918(51)90007-8

[B23] Rudin-Brown C. (2004). Vehicle height affects drivers' speed perception implications for rollover risk. Transp. Res. Rec. 1899, 84–89 10.3141/1899-11

[B24] Salvucci D. D. (2009). Rapid prototyping and evaluation of in-vehicle interfaces. ACM Transactions on Computer-Human Interaction 16, 9 10.1145/1534903.1534906

[B25] Shi Z. Church R. M. Meck W. H. (2013). Bayesian optimization of time perception. Trends Cogn. Sci. 17, 556–564 10.1016/j.tics.2013.09.009 24139486

[B26] Taatgen N. A. van Rijn H. Anderson J. (2007). An integrated theory of prospective time interval estimation: the role of cognition, attention, and learning. Psychol. Rev. 114, 577–598 10.1037/0033-295X.114.3.577 17638497

[B27] Taatgen N. van Rijn H. (2011). Traces of times past: representations of temporal intervals in memory. Mem. Cogn. 39, 1546–1560 10.3758/s13421-011-0113-0 21626068 PMC3205264

[B28] Tse P. U. Intriligator J. Rivest J. Cavanagh P. (2004). Attention and the subjective expansion of time. Percept. Psychophys. 66, 1171–1189 10.3758/BF03196844 15751474

[B29] van Rijn H. Gu B.-M. Meck W. H. (2014). Dedicated clock/timing-circuit theories of time perception and timed performance, in Neurobiology of Interval Timing, eds Merchant H. de Lafuente V. (New York, NY: Springer-Verlag).

[B30] van Rijn H. Kononowicz T. W. Meck W. H. Ng K. K. Penney T. B. (2011). Contingent negative variation and its relation to time estimation: a theoretical evaluation. Front. Integr. Neurosci. 5:91 10.3389/fnint.2011.00091 22207841 PMC3246349

[B31] van Rijn H. Taatgen N. A. (2008). Timing of multiple overlapping intervals: how many clocks do we have? Acta Psychol. (Amst). 129, 365–375 10.1016/j.actpsy.2008.09.002 18849020

[B32] van Wassenhove V. Buonomano D. V. Shimojo S. Shams L. (2008). Distortions of subjective time perception within and across senses. PLoS ONE 3:e1437 10.1371/journal.pone.0001437 18197248 PMC2174530

[B33] Wiener M. Kliot D. Turkeltaub P. E. Hamilton R. H. Wolk D. A. Coslett H. B. (2012). Parietal influence on temporal encoding indexed by simultaneous transcranial magnetic stimulation and electroencephalography. J. Neurosci. 32, 12258–12267 10.1523/JNEUROSCI.2511-12.2012 22933807 PMC3448365

[B34] Wittmann M. (2013). The inner sense of time: how the brain creates a representation of duration. Nat. Rev. Neurosci. 14, 217–223 10.1038/nrn3452 23403747

[B35] Wittmann M. van Wassenhove V. Craig A. D. Paulus M. P. (2010). The neural substrates of subjective time dilation. Front. Hum. Neurosci. 4, 2 10.3389/neuro.09.002.2010 20161994 PMC2820380

